# Whole-genome characterization of a colistin-resistant *Klebsiella quasipneumoniae subsp. quasipneumoniae* isolate from a urinary tract infection in Peshawar, Pakistan

**DOI:** 10.1007/s42770-026-01982-0

**Published:** 2026-06-10

**Authors:** Aiman Waheed, Sumera Afzal Khan, Sajjad Ahmad, Taj Ali Khan, Taane G. Clark

**Affiliations:** 1 Center of Biotechnology and Microbiology, University of Peshawar, Peshawar, Pakistan; 2 Institute of Pathology and Diagnostic Medicine (IPDM), Khyber Medical University, Peshawar, Pakistan; 3 Public Health Reference Laboratory, Khyber Medical University, Peshawar, Pakistan; 4 Faculty of Infectious and Tropical Diseases, London School of Hygiene and Tropical Medicine, London, UK; 5 Faculty of Epidemiology and Population Health, London School of Hygiene and Tropical Medicine, London, UK

**Keywords:** *K. pneumoniae* species complex, *Klebsiella* quasipneumoniae, Colistin resistance, Whole-genome sequencing, Chromosomal antimicrobial resistance, Multidrug resistance, Urinary tract infection, Genomic surveillance

## Abstract

*Klebsiella quasipneumoniae* is a Gram-negative, non-motile, capsulated, facultative anaerobic rod within the *K. pneumoniae* species complex (KpSC) and is increasingly recognized as an opportunistic pathogen associated with bloodstream infections, urinary tract infections (UTIs), and other clinically significant conditions. Because it shares many phenotypic characteristics with *K. pneumoniae*, accurate diagnosis remains challenging in routine clinical settings, particularly in low-resource laboratories lacking access to molecular identification tools. In this study, we characterized the antimicrobial resistance (AMR) profile of a *K. quasipneumoniae subsp. quasipneumoniae* isolate obtained from a UTI case in Peshawar, Pakistan. The isolate exhibited a multidrug resistant (MDR) phenotype, with resistance to β-lactams, carbapenems, fluoroquinolones, and colistin (MIC 4 µg/mL), while remaining susceptible to aminoglycosides (amikacin and gentamicin) and tigecycline (MIC 2 µg/mL). Whole-genome sequencing (WGS) identified chromosomally encoded AMR determinants, including *bla*OKP-A-8, *oqxAB*, and fosA6, with no identifiable plasmid replicons detected. Multiple nonsynonymous mutations were observed in *mgrB*,* pmrA/B*,* phoP/Q*,* lpxM*,* ompK35/36*, and *gyrA/parC*, which have been previously associated with resistance phenotypes; however, their functional contribution in this isolate was inferred from genomic data and not experimentally validated. Virulence-associated loci such as *fim*,* ecp*,* entB*, and *fepC* were present, consistent with a classical (non-hypervirulent) phenotype. Phylogenomic analysis positioned the isolate (Kq1223) on a distinct branch relative to publicly available genomes, but given that this study is based on a single isolate, no definitive conclusions regarding regional lineage or evolutionary patterns can be established. The allelic profile identified by multilocus sequence typing (MLST) has not been previously reported and may represent an unassigned sequence type pending formal database validation. The presence of chromosomally mediated MDR, including colistin resistance, highlights potential therapeutic challenges and underscores the importance of accurate species identification and expanded genomic surveillance of *Klebsiella* species in clinical microbiology, particularly in resource limited settings.

**Supplementary Information:**

The online version contains supplementary material available at 10.1007/s42770-026-01982-0.

## Introduction

Over recent decades, advances in modern healthcare including widespread antibiotic use, prolonged hospital stays, and an increasing number of medical procedures have inadvertently created ecological niches that favor the emergence and spread of MDR and extensively drug resistant (XDR) pathogens. Opportunistic organisms such as *Acinetobacter baumannii* and *Candida auris*, once rarely encountered in clinical settings, have evolved into major nosocomial threats with remarkable resistance to multiple antimicrobial classes [1]. Among these, *K. pneumoniae*, a clinically significant member of the ESKAPE pathogen group, has become a leading cause of both nosocomial and community acquired infections, including pneumonia, urinary tract infections, bacteremia, and neonatal sepsis. Importantly, carbapenems resistant and extended spectrum cephalosporin resistant *K. pneumoniae* strains are recognized by the World Health Organization (WHO) as critical priority pathogens because of limited therapeutic options and high associated morbidity and mortality [2, 3]. Although part of the normal microbiota of the gastrointestinal tract and skin, *K. pneumoniae* can cause severe and life-threatening disease, particularly in neonates, the elderly, and immunocompromised individuals [4].

The pathogenicity of *K. pneumoniae* is driven by a range of virulence determinants, including its polysaccharide capsule, siderophore-mediated iron acquisition systems, adhesins, and regulatory genes such as *rmpA*, which contributes to the hypermucoviscous phenotype [5]. Over the past two decades, the emergence of hypervirulent *K. pneumoniae* (hvKp) lineages has further reshaped its clinical and epidemiological landscape, with strains capable of causing invasive syndromes such as pyogenic liver abscess and meningitis even in otherwise healthy individuals [5]. Concurrently, WGS and phylogenomic studies have revealed that *K. pneumoniae* is part of a broader clade, the KpSC, which includes *K. pneumoniae sensu stricto* (KpI), *K. quasipneumoniae* (KpII), and *K. variicola* (KpIII), among others closely related taxas. Members of KpSC are frequently misidentified by conventional phenotypic methods, resulting in the underestimation of species-specific epidemiology, virulence characteristics, and AMR patterns [6, 7]. Within the KpSC, *K. quasipneumoniae* has diverged into two genetically distinct subspecies *K. quasipneumoniae* subsp. *quasipneumoniae* (KpII-A/Kp2) and *K. quasipneumoniae* subsp. *similipneumoniae* (KpII-B/Kp4) both of which remain phenotypically similar to *K. pneumoniae* [8]. Accurate differentiation therefore relies on molecular approaches such as multilocus sequence typing, average nucleotide identity, or WGS-based classification [9, 10]. The species name *quasipneumoniae* (“almost pneumoniae”) reflects its close genetic proximity, which historically led to widespread misidentification by routine clinical microbiology platforms such as API 20E, VITEK, and MALDI-TOF MS [11, 12]. Accurate species differentiation now relies on molecular or genomic tools, including multilocus sequence typing (MLST) and WGS [13, 14].

Although *K. quasipneumoniae* was initially regarded as an environmental species, it is increasingly recognized as a clinically meaningful pathogen isolated from bloodstream, urinary, and wound infections, particularly among immunocompromised patients [15, 16]. Recent studies demonstrate its capacity to acquire and disseminate extended-spectrum β-lactamase (ESBL) and carbapenemase genes - including *blaNDM*, *blaKPC*, and *blaOXA-48* - through horizontal gene transfer, positioning it as a potential reservoir of MDR determinants within the KpSC [17–20]. However, the extent to which chromosomal versus plasmid-mediated mechanisms contribute to resistance in this species remains incompletely understood [21]. MDR *Klebsiella* species have also been identified in hospital wastewater and environmental reservoirs, which may facilitate the exchange of mobile genetic elements and contribute to the persistence of resistance determinants in healthcare settings [22].

Colistin remains a last-line therapeutic option for MDR Gram-negative infections; however, resistance to this agent is rising, driven either by chromosomal mutations (e.g., *mgrB*, *pmrAB*, *phoPQ*, *lpxM*) or by plasmid-mediated *mcr* genes [23, 24]. While colistin resistance has been extensively documented in *K. pneumoniae sensu stricto*, it remains comparatively underreported in *K. quasipneumoniae*, and the underlying molecular mechanisms are not fully elucidated [25, 26]. Furthermore, clinical management of MDR Gram negative infections in low-resource settings is complicated by limited diagnostic capacity and restricted access to effective antimicrobials [27]. In Pakistan, carbapenems resistant *K. pneumoniae* carrying *bla*NDM and *bla*OXA-48 are well documented [28, 29], however, genomic data on *K. quasipneumoniae*, particularly from Peshawar, remain extremely limited [19].

In this context, the present study provides a WGS based characterization of a colistin non-susceptible *K. quasipneumoniae subsp. quasipneumoniae* isolate (Kq1223) recovered from a UTI patient in Peshawar, Pakistan. Given that the analysis is based on a single clinical isolate, the findings should be interpreted as exploratory and not necessarily representative of broader epidemiological trends. Using WGS and comparative genomic approaches, we describe its AMR determinants, virulence associated loci, and phylogenetic relationships of Kq1223 in comparison with publicly available reference genomes. Collectively, these findings provide preliminary genomic insights into *K. quasipneumoniae* circulating in an underrepresented geographic region and underscore the need for expanded genomic surveillance, larger scale epidemiological investigations, and functional studies to further elucidate resistance mechanisms, pathogenic potential, and clinical implications associated with this emerging species.

## Materials and methods

### Ethical approval

The study was approved by the Research Ethics Board of the University of Peshawar, Pakistan (Approval No. REB-10). All patients were informed about the study procedures, and written informed consent was obtained prior to sample collection.

### Sample collection

A total of 600 clinical samples, including blood, urine, sputum, pus, wound swabs, and other body fluids, were collected between August 2024 and July 2025 from three tertiary care hospitals in Peshawar, Pakistan: Lady Reading Hospital (LRH), Khyber Teaching Hospital (KTH), and Hayatabad Medical Complex (HMC). All samples were labelled and transported to the Microbiology Laboratory at the Centre of Biotechnology and Microbiology for further processing. Initial bacterial identification was performed using standard microbiological procedures, including Gram staining, assessment of colony morphology, and biochemical characterisation with the API 20E system (bioMérieux SA, Lyon, France). Based on these preliminary analyses, 64 isolates were identified as *Klebsiella* spp., matching the reference profile for *K. pneumoniae* (catalogue number 5214573) [30]. Consistent with the recognized limitations of phenotypic methods for distinguishing members of the KpSC, isolates selected for further investigation were subsequently subjected to molecular confirmation.

### Antimicrobial susceptibility testing

Antimicrobial susceptibility testing (AST) of all 64 Klebsiella isolates was performed using the Kirby–Bauer disk diffusion method on Mueller–Hinton agar (Oxoid, UK), in accordance with Clinical and Laboratory Standards Institute (CLSI) 2023 guidelines [30]. Bacterial suspensions were standardized to 0.5 McFarland turbidity and inoculated uniformly onto agar plates. The antimicrobial agents tested included amikacin (30 µg), amoxicillin–clavulanic acid (20/10 µg), cefotaxime (30 µg), ceftazidime (30 µg), cefepime (30 µg), cefoperazone–sulbactam (75/30 µg), piperacillin–tazobactam (100/10 µg), ceftazidime–avibactam (30/20 µg), meropenem (10 µg), ciprofloxacin (5 µg), tetracycline (30 µg), and gentamicin (10 µg). Plates were incubated at 37 °C for 18–24 h, and inhibition zone diameters were interpreted according to CLSI breakpoints.

Additional carbapenems (e.g., imipenem, ertapenem) were not available within the routine testing panel during the study period. Given the recognized limitations of disk diffusion for certain agents, susceptibility to tigecycline and colistin was determined using broth microdilution (ComASP Colistin, Liofilchem, Italy) and Etest, with minimum inhibitory concentrations (MICs) interpreted according to CLSI criteria. For the purpose of resistance classification, isolates categorized as either intermediate or resistant were considered non-susceptible, consistent with international consensus definitions [31]. Antimicrobial agents were grouped into epidemiologically relevant categories for Enterobacterales based on the framework proposed by Magiorakos et al. (2012) MDR, XDR, and PDR definitions, including aminoglycosides, fluoroquinolones, carbapenems, extended-spectrum cephalosporins, β-lactam/β-lactamase inhibitor combinations, tetracyclines, polymyxins, and glycylcyclines [32]. Antimicrobial resistance phenotypes met operational criteria consistent with MDR/XDR definitions based on the tested antimicrobial categories [32].

### Species identification and WGS

For genomic analysis, 25 Klebsiella isolates were selected for WGS, comprising 13 MDR and 12 XDR isolates. Selection was based primarily on antimicrobial resistance profiles, with priority given to isolates exhibiting MDR or XDR phenotypes in order to maximize detection of clinically relevant resistance determinants. Only isolates yielding high-quality genomic DNA, as determined by DNA integrity and purity assessment, were included for sequencing. Genomic DNA was extracted using the GenJet Genomic DNA Purification Kit (Thermo Fisher Scientific, USA) according to the manufacturer’s instructions. DNA quality and integrity were evaluated by agarose gel electrophoresis, while DNA concentration and purity were measured using a NanoDrop spectrophotometer (Thermo Fisher Scientific, USA). Sequencing libraries were prepared using the QIAseq FX DNA Library Kit (Qiagen, Germany) and sequenced on the Illumina MiSeq platform using a 150-bp paired-end protocol, targeting approximately 100× genome coverage [33].

### Bioinformatic analysis

Raw sequencing read quality was assessed using FastQC (v0.11.9). Adapter removal and trimming of low-quality bases were performed with Fastp (v0.23.2), applying a Phred quality threshold of Q30 and a minimum read length of 50 bp [34]. De novo genome assembly was carried out using Shovill (v1.1.0) with SPAdes as the underlying assembler. Assembly metrics including genome length, GC content, N50, and contig number were evaluated using QUAST [35]. Genome annotation was performed with Prokka (v1.14.6), and annotations were cross-validated using the Rapid Annotation using Subsystem Technology (RAST) server [34]. Taxonomic assignment was conducted with Kraken, and outputs were formatted using ConvertKraken [36]. To ensure accurate species level identification within the KpSC, Average Nucleotide Identity (ANI) analysis was performed using FastANI (v1.3) against representative reference genomes *Klebsiella quasipneumoniae subsp. quasipneumoniae* strain 01A030T (NZ_CP084876.1) and *Klebsiella pneumoniae subsp. pneumoniae* HS11286 (CP003200.1), including type strains. Species delineation was interpreted using the accepted ANI threshold of ≥ 95–96% [37]. Among all sequenced isolates, only one designated Kq1223 was confirmed as *K. quasipneumoniae* subsp. *quasipneumoniae* and selected for in-depth genomic characterization. Identification and visualization of Clustered Regularly Interspaced Short Palindromic Repeats (CRISPR) loci were performed using CRISPRFinder [38].

### AMR and virulence gene profiling

The AMR gene profile was identified using ResFinder and the Comprehensive Antibiotic Resistance Database (CARD) [39]. Chromosomal resistance–associated point mutations were confirmed using AMRFinderPlus [40]. Virulence factors were detected through the Virulence Factor Database (VFDB) [41]. MLST was performed to determine the sequence type (ST) of the isolate within the KpSC [34]. Plasmid replicons were screened using PlasmidFinder [42].

### Phylogenetic and statistical analysis

**Fig. 1:**
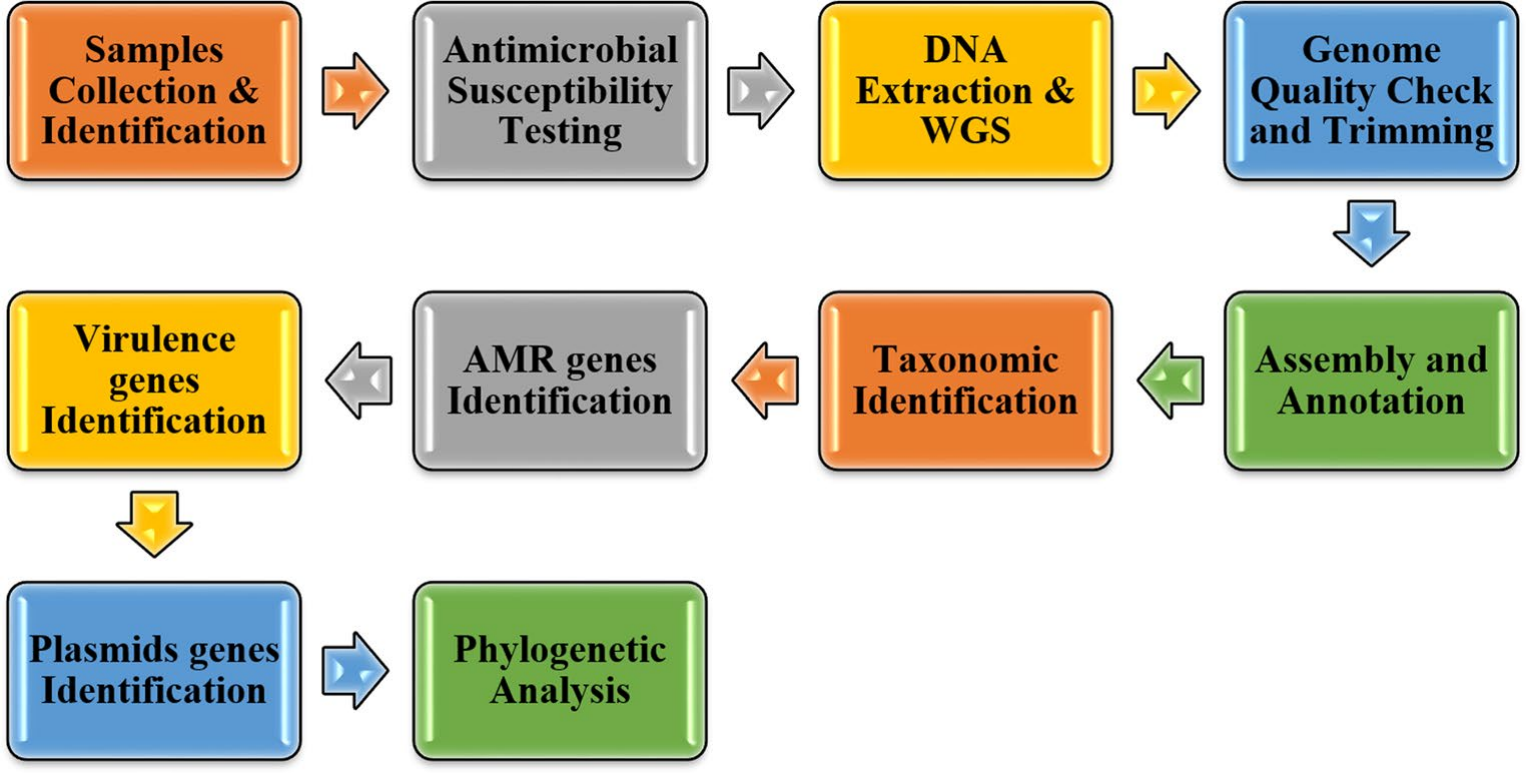
Methodology flow chart

The phylogenetic placement of isolate Kq1223 was determined using the Bacterial and Viral Bioinformatics Resource Center (BV-BRC) Bacterial Genome Tree Service. A codon-based tree was generated using 100 single-copy core genes conserved across all representative *K. quasipneumoniae* genomes available in the BV-BRC database [43]. Reference genomes were selected based on availability within the BV-BRC database, and included all publicly available high quality WGS of *K. quasipneumoniae subsp. quasipneumoniae* isolated from human samples at the time of analysis. The resulting Newick file was reconstructed in MEGA11, and the phylogeny was visualized using Interactive Tree of Life (iTOL) to assess the evolutionary relationship between Kq1223 and other members of the *K. quasipneumoniae* clade [44]. Statistical analyses, including frequency distributions of resistance phenotypes, were performed in STATA statistical software to provide an overview of resistance patterns among the isolates (Fig. 1).

## Results

### Clinical and demographic profile of the study cohort

A total of 64 confirmed *Klebsiella* isolates were collected from patients admitted to three tertiary care hospitals (KTH, HMC, and LRH) in Peshawar, Pakistan, between August 2024 and July 2025. The median patient age was 57 years (interquartile range: 45–68), and 62.5% were male. Most patients (71.9%) belonged to the Pashtun ethnic group, predominantly residing in the northern districts of Peshawar, Mardan, and Swabi. Common comorbidities included type 2 diabetes mellitus (34.4%), chronic kidney disease (21.9%), and hypertension (20.3%). The most frequent sites of infection were skin and soft tissue (34.4%), wounds (28.1%), and the urinary tract (14.1%), followed by abscesses (10.9%). Bloodstream infections were less common (4.7%) and occurred mainly among patients with multiple or severe underlying conditions (Table S1). These demographic and clinical characteristics represent the overall Klebsiella isolate cohort and are not specific to the *K. quasipneumoniae* isolate Kq1223.

### Antibiotic susceptibility profiles

**Fig. 2:**
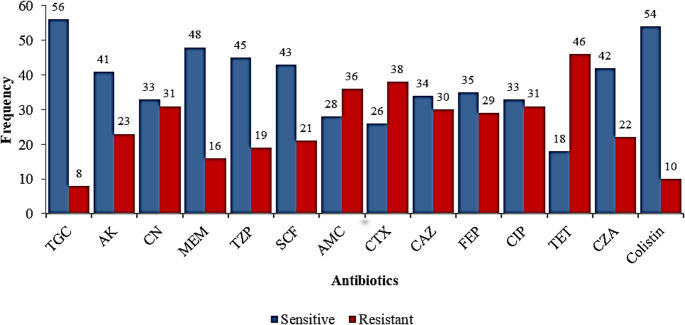
Antibiotic resistance frequencies in the *Klebsiella* isolates (*n* = 64). The antibiotics are: amikacin (AK), amoxicillin–clavulanic acid (AMC), cefotaxime (CTX), ceftazidime (CAZ), ciprofloxacin (CIP), cefepime (FEP), cefoperazone–sulbactam (SCF), piperacillin–tazobactam (TZP), ceftazidime–avibactam (CZA), tigecycline (TGC), gentamicin (CN), tetracycline (TET), and meropenem (MEM)

Among the 64 clinical *Klebsiella* isolates analysed, 22 isolates (34.4%) were categorized as MDR and 12 isolates (18.8%) as XDR, based on the operational application of standardized definitions proposed by Magiorakos et al. (2012). No isolates fulfilled the criteria for PDR. However, because the antimicrobial susceptibility panel did not include all antimicrobial categories recommended for Enterobacterales, these classifications should be considered provisional. Tigecycline and colistin were the most effective antibiotics, with susceptibility rates of 87.5% and 84.37%, respectively. Among the aminoglycosides, amikacin demonstrated higher activity (64.1%) compared with gentamicin (51.6%). Meropenem was effective against 75% of isolates, indicating moderate carbapenem susceptibility. Combination β-lactam/β-lactamase inhibitor agents’ piperacillin-tazobactam (70.3%) and cefoperazone-sulbactam (67.2%) also showed favourable activity. Cephalosporins exhibited variable performance: cefotaxime had low activity (40.6%), while ceftazidime (53.1%) and cefepime (54.7%) showed intermediate susceptibility rates. Ceftazidime–avibactam was active against 65.6% of isolates, although resistance exceeded one-third of cases. Ciprofloxacin (51.6%) showed limited efficacy, and tetracycline had the lowest activity (28.1%) (Fig. 2).

### Klebsiella lineage identification via WGS

WGS was performed on 25 isolates belonging to the KpSC. Phylogenomic analysis identified 24 isolates as *K. pneumoniae subsp. pneumoniae*, whereas one isolate displayed a distinct taxonomic profile. ANI analysis of this isolate, designated Kq1223, demonstrated 99.02% identity with *K. quasipneumoniae subsp. quasipneumoniae* strain 01A030T and 93.84% identity with *K. pneumoniae subsp. pneumoniae* HS11286. Based on established ANI thresholds for species delineation, the isolate was conclusively assigned to *K. quasipneumoniae subsp. quasipneumoniae* (Table 1; Figure S1).

**Table 1:** Average nucleotide identity analysis of isolate Kq1223 against reference genomes

Query isolate	Reference genome	Accession number	ANI (%)	Bidirectional Fragment Mappings	Total Query Fragments	Taxonomic interpretation
Kq1223	*K. quasipneumoniae* subsp. *quasipneumoniae* strain 01A030T	NZ_CP084876.1	99.02	1639	1769	Same species
Kq1223	*K. pneumoniae* subsp. *pneumoniae* HS11286	CP003200.1	93.84	1508	1769	Different species

As the present study is based on a single *K. quasipneumoniae* isolate, the findings should be interpreted as descriptive rather than representative of broader population structure or regional epidemiology. Nevertheless, given the limited genomic data available for this species from Pakistan, isolate Kq1223 was selected for comprehensive genomic characterization to provide preliminary insights into the diversity of the KpSC in this underrepresented geographic region.

### Clinical and microbiological characteristics of Kq1223

The patient from whom Kq1223 was isolated was a 56-year-old female with a medical history of type 2 diabetes mellitus, hypertension, and chronic kidney disease. She presented to the hospital with fever, burning micturition, and shortness of breath, and provided a urine sample that was subsequently diagnosed as a UTI. AST revealed that Kq1223 was non-susceptible to meropenem, piperacillin-tazobactam, cefoperazone-sulbactam, amoxicillin-clavulanate, cefotaxime, ceftazidime, cefepime, ciprofloxacin, tetracycline, ceftazidime-avibactam, and colistin (Table 2). Susceptibility to colistin and tigecycline was determined by broth microdilution (MIC = 4 µg/mL for colistin and 2 µg/mL for tigecycline). Given the resistance profile across multiple antimicrobial categories, Kq1223 meets the operational criteria for MDR.

**Table 2:** Clinical, microbiological, and AST profile of Kq1223

Parameter	Result / Interpretation
Patient demographics	56-year-old female, Peshawar, Pakistan
Clinical background	Type 2 diabetes mellitus, hypertension, chronic kidney disease
Clinical presentation	Fever, burning micturition, shortness of breath
Key laboratory findings	CRP: 86 mg/L; WBC: 11.2 × 10³/µL; Hb: 9.1 g/dL; HCT: 28.7%; PLT: 110 × 10³/Μl
Sample type	Urine
Culture findings	Mucoid lactose-fermenting colonies (> 10⁵ CFU/mL) on blood agar and CLED agar
Gram staining	Gram-negative bacilli
API 20E identification	*K. pneumoniae*
WGS	*K. quasipneumoniae* subsp. *Quasipneumoniae*
Genome features	Chromosome-only; no plasmid replicons detected
Colistin MIC (µg/mL)	4 (Resistant, CLSI 2023)
Sensitive to	Tigecycline, Amikacin, Gentamicin
Resistant to	Meropenem, Piperacillin-tazobactam, Cefoperazone-sulbactam, Amoxicillin-clavulanate, Cefotaxime, Ceftazidime, Cefepime, Ciprofloxacin, Tetracycline, and Ceftazidime-avibactam

### Genome features of Kq1223

Analysis of the WGS data showed that Kq1223 had a genome size of 5.37 Mb and a GC content of 57.95%. The assembly consisted of 98 contigs with an N50 of 365,211 bp and an L50 of 5, indicating a high-quality draft genome. Genome annotation identified 5,227 coding sequences, 86 tRNA genes, and 13 rRNA operons (Table S2). MLST produced the following allelic profile: *gapA* [17], *infB* [19], *mdh* (156), *pgi* (331), *phoE* (207), *rpoB* [21], and *tonB* (589). This allelic profile has not been previously reported in current MLST databases and is therefore considered unassigned, pending formal submission and validation; no definitive designation of a novel lineage can be made at this stage.

### AMR determinants

Genomic analysis of isolate Kq1223 revealed a restricted yet characteristic resistome consisting solely of four chromosomally encoded intrinsic resistance genes: *blaOKP-A-8_1*,* oqxA_1*,* oqxB_1*, and *fosA6_1*. The *blaOKP-A-8_1*gene encodes an OKP-type β-lactamase that provides minimal resistance to penicillins and is not an extended-spectrum enzyme [45]. The *oqxA* and *oqxB* genes encode the OqxAB efflux pump, which contributes to intrinsic resistance to fluoroquinolones, chloramphenicol, and several disinfectants [46]. The *fosA6_1* gene encodes a glutathione transferase that confers low-level resistance to fosfomycin [47]. No known plasmid replicons or acquired antimicrobial resistance genes were detected using PlasmidFinder and ResFinder; however, this does not exclude the presence of uncharacterized or cryptic mobile genetic elements. Therefore, antimicrobial resistance in Kq1223 appears to be predominantly chromosomally encoded, although the absence of horizontal gene transfer cannot be definitively established.

Analysis of resistance-associated chromosomal loci identified multiple nonsynonymous substitutions in genes linked to colistin, carbapenem, and fluoroquinolone resistance (Table 3). Mutations in *pmrA/pmrB*,* phoQ*,* mgrB*, and *lpxM* are consistent with mechanisms reported to confer colistin resistance, likely through lipid A remodeling and reduced drug–lipid A binding affinity. Alterations in *ompK35* and *ompK36* are consistent with reduced outer membrane permeability, which has been associated with decreased carbapenem susceptibility. Additionally, mutations in *gyrA* and *parC* aligned with fluoroquinolone resistance by altering DNA gyrase and topoisomerase IV function. It is important to emphasize that these genotype–phenotype associations are inferred from genomic data and existing literature, and no functional validation (e.g., complementation or expression analysis) was performed in this study.

**Table 3:** Key chromosomal mutations associated with antimicrobial resistance in Kq1223. All listed amino acid substitutions were detected within the genome of the single isolate Kq1223 and do not represent alternative allelic variants observed across multiple isolates. The predicted functional significance of each mutation is based on previously reported associations with antimicrobial resistance mechanisms

Gene	Key Mutation(s)	Antibiotic Class Affected	Functional/Clinical Significance
*PmrA/PmrB*	E199D, D216Y, A228T, Q232E, Q356R	Colistin	Alters sensor-regulator signaling, enhancing lipid A modification and resistance to colistin.
*PhoQ*	A106T, E112D, Q424P, Q482L	Colistin	Modifies lipid A synthesis regulation; associated with decreased colistin binding.
*MgrB*	M1V	Colistin	Inactivates negative regulator of PhoPQ, conferring stable colistin resistance.
*LpxM*	F320Y, K290T, Y80H	Colistin	Affects lipid A acylation, contributing to membrane charge alteration.
*OmpK35/OmpK36*	A182S, D338A, H349R, V178P	Carbapenem	Reduces outer membrane permeability, limiting carbapenem influx.
*GyrA/ParC*	D445E, T408A, G408A, S438N	Fluoroquinolone	Substitutions in quinolone-resistance–determining regions (QRDRs) decrease fluoroquinolone binding affinity.

### Virulence determinants

Virulence gene screening revealed a conserved but limited virulence repertoire in Kq1223. Core determinants included *ompA*, *entB*, and *fepC*, which are involved in adhesion and iron acquisition. The presence of *ykgK/ecpR* and *yagZ/ecpA*, encoding components of the *E. coli* common pilus, suggests a potential for moderate adherence and biofilm formation. No hypervirulence-associated loci (e.g., *rmpA*,* iuc*,* ybt*) were identified, supporting classification of Kq1223 as a classical (non-hypervirulent) opportunistic strain.

### Phylogenetic positioning

**Fig. 3:**
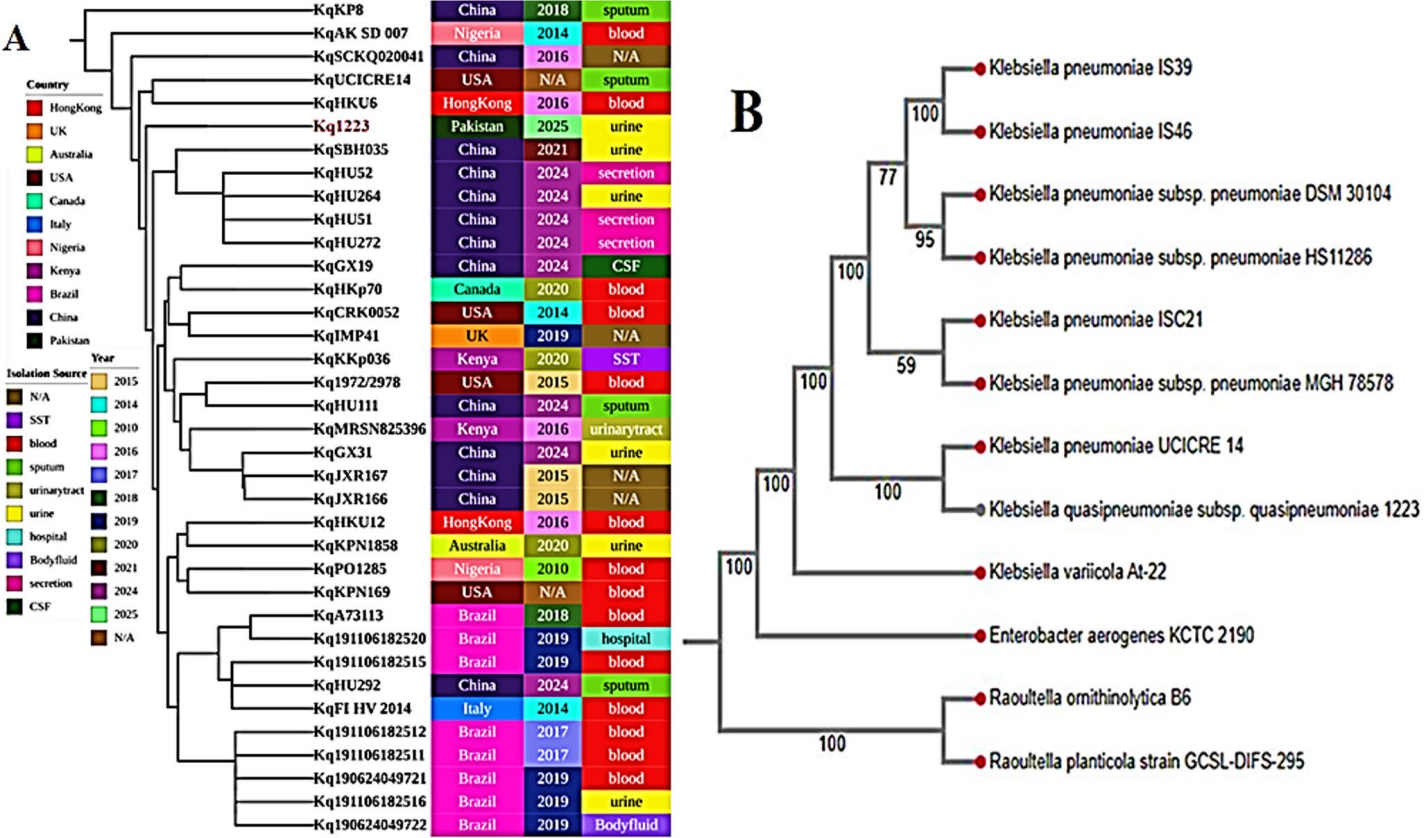
(**A**) Whole-genome phylogenetic tree of *Klebsiella quasipneumoniae* isolates, based on 35 publicly available genomes collected from nine countries between 2010 and 2024. The top row indicates the country of isolation, the middle row shows the year of collection, and the bottom row denotes the sample source. (**B**) Phylogenetic tree depicting the relationship of *Klebsiella quasipneumoniae* subsp. *quasipneumoniae* strain Kq1223 with representative members of the *K. pneumoniae* species complex (KpSC)

Whole-genome phylogenetic analysis placed the Kq1223 isolate within the *K. quasipneumoniae* subsp. *quasipneumoniae* lineage (Fig. 3A, Figure S2). The genomes included in the phylogenomic analysis, along with their accession numbers, isolation sources, collection years, bioproject number, and countries of origin, are summarized in Table S3. The isolate formed a distinct branch most closely related to HKU6 (Hong Kong) and UCICRE14 (USA). However, given that this analysis is based on a single isolate, this observation should not be interpreted as evidence of a distinct regional lineage or evolutionary trajectory. Rather, Kq1223 clusters separately from currently available genomes, which may reflect undersampling of this species in South Asia and highlights the need for expanded genomic surveillance. Kq1223 represents one of the first whole genome sequenced *K. quasipneumoniae* isolates reported from Pakistan, thereby contributing to the currently limited genomic data from this region. Within the broader phylogeny of the KpSC (Fig. 3B), Kq1223 clustered within the *K. quasipneumoniae* subsp. *quasipneumoniae* clade and was clearly separated from *K. pneumoniae sensu stricto* and *K. variicola*. This placement, supported by 100% bootstrap values, confirms the isolate’s precise taxonomic identity.

### CRISPR-Cas system

**Fig. 4:**
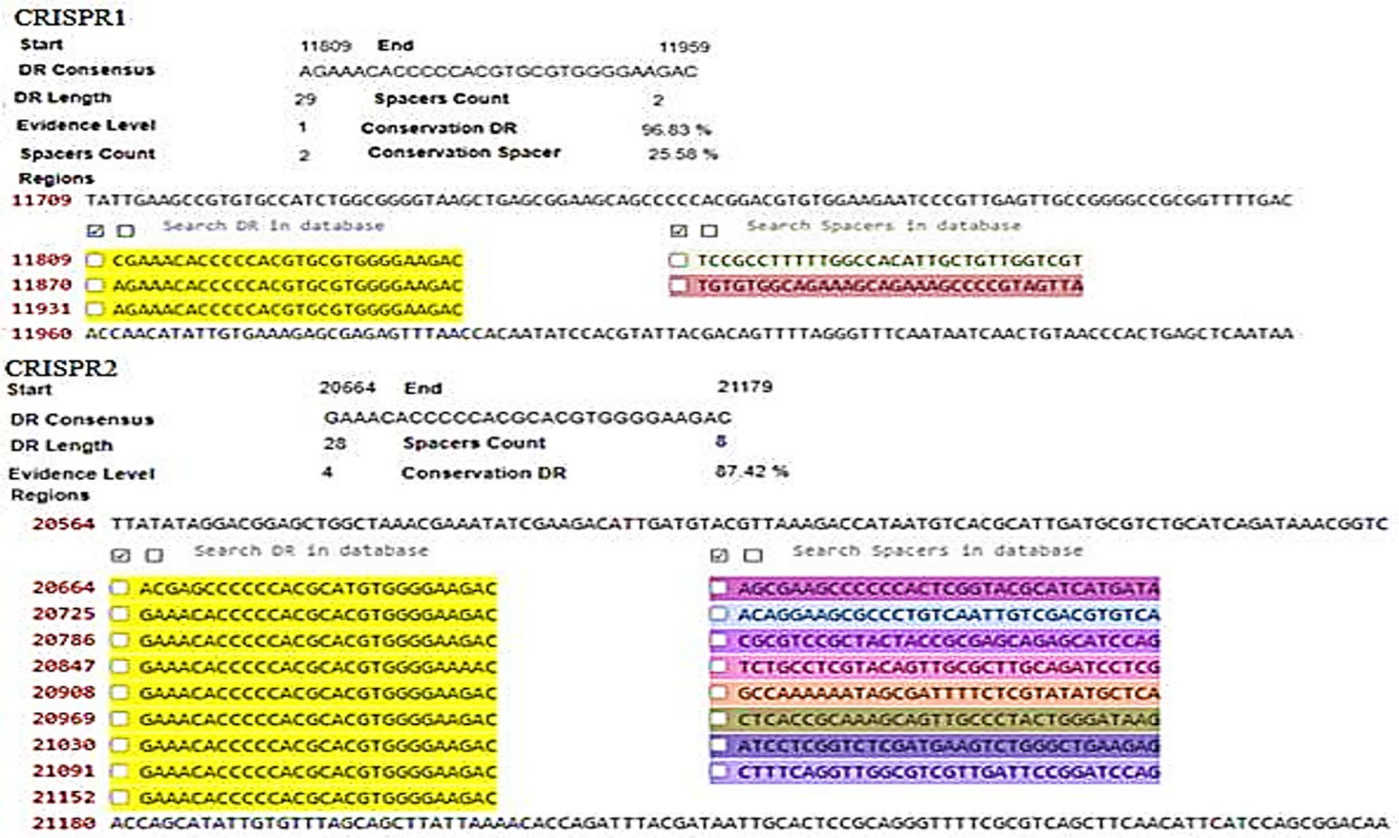
Organization of the CRISPR–Cas system in *Klebsiella quasipneumoniae* subsp. *quasipneumoniae* strain Kq1223. Two CRISPR arrays (CRISPR1 and CRISPR2) were identified, each associated with a complete Type I-E Cas gene cluster

Two distinct CRISPR arrays were identified in the Kq1223 genome. CRISPR1 (positions 11,809–11,959 bp) comprised two spacers with 96.8% repeat conservation, while CRISPR2 (positions 20,664–21,179 bp) contained eight spacers with 87.4% repeat conservation. Both arrays were associated with a complete Type I-E CRISPR–Cas system (cas1–cas7, cse1–cse2, cas3), suggesting that Kq1223 possesses an active adaptive immune mechanism against invading genetic elements (Fig. 4). Although the presence of a complete CRISPR–Cas system may contribute to restriction of foreign DNA acquisition, no spacer–protospacer matching analysis was performed; therefore, the functional activity of the CRISPR-Cas system remains undetermined.

## Discussion

*K. quasipneumoniae* was recognized as a distinct member of the *KpSC* in 2014, yet it remains underreported and poorly understood despite its increasing clinical relevance [11]. Initially regarded as an environmental or low virulence species, accumulating evidence now implicates *K. quasipneumoniae subsp. quasipneumoniae* in urinary tract, bloodstream, biliary, and respiratory infections, particularly among hospitalized and immunocompromised patients [16, 48]. However, genomic data for this subspecies remain limited globally, with most available studies originating from China, Brazil, and the United States [7, 11, 49]. Consequently, the present study provides a single isolate; whole genome based characterization from an underrepresented geographic region and should be interpreted as a descriptive genomic report rather than a population-level epidemiological analysis.

WGS confirmed isolate Kq1223 as *K. quasipneumoniae subsp. quasipneumoniae*, highlighting the limitations of conventional phenotypic identification methods such as API 20E, which frequently fail to distinguish members of the KpSC [7, 50]. Phenotypically, isolate Kq1223 produced mucoid lactose-fermenting colonies and displayed biochemical profiles typical of *K. pneumoniae* [51]. These findings reinforce the importance of genomic approaches, including ANI analysis and WGS-based taxonomic classification, for accurate differentiation of closely related Klebsiella species in clinical microbiology laboratories [52].

Genomic analysis demonstrated that no known plasmid replicons or acquired antimicrobial resistance genes were detected in Kq1223, and the resistome appeared predominantly chromosomally encoded. However, the absence of detectable plasmids using currently available bioinformatic tools does not exclude the presence of uncharacterized, cryptic, or low-copy extrachromosomal elements. The resistance genes included *blaOKP-A-8*, *fosA6*, and the *oqxA/B* efflux system, conferring resistance to β-lactams, fosfomycin, and quinolones, respectively. Unlike plasmid-mediated β-lactamases such as *blaSHV* or *blaCTX-M* in *K. pneumoniae*, *blaOKP-A-8* is chromosomal and specific to *K. quasipneumoniae* [53, 54]. Importantly, the resistance determinants identified in Kq1223 largely represent previously recognized chromosomal mechanisms rather than unusual or highly divergent genomic features, and no acquired carbapenemase or transferable colistin resistance genes were detected [54, 55].

Phenotypically, Kq1223 was not susceptible to β-lactams, carbapenems, fluoroquinolones, tetracyclines, and colistin, but remained susceptible to aminoglycosides and tigecycline. Multiple nonsynonymous substitutions were identified in *pmrA/pmrB*,* phoQ*,* mgrB*, and *lpxM* all of which have previously been associated with colistin resistance through lipid A modification and altered outer membrane charge. Some of the observed substitutions correspond to mutations previously described in Klebsiella spp., whereas others have not been extensively reported in K. quasipneumoniae and therefore remain putative resistance-associated variants. Notably, no plasmid-mediated *mcr* genes were detected, suggesting that colistin non-susceptibility in Kq1223 is likely independent of known transferable resistance determinants [7, 24, 56, 57]. Nevertheless, the contribution of these mutations to the observed resistance phenotype was inferred from genomic data and published literature and was not experimentally validated. No complementation assays, transcriptional analyses, lipid A characterization, or gene expression studies were performed; therefore, direct causality between the identified mutations and colistin resistance cannot be conclusively established. Additional mechanisms, including efflux activity, regulatory pathway alterations, or capsule-associated effects, may also contribute to reduced colistin susceptibility [51].

Similarly, reduced carbapenem susceptibility was associated with alterations in outer membrane porins (*ompK35* and *ompK36*), which are known to reduce permeability to β-lactams [58, 59], while fluoroquinolone resistance was linked to substitutions in *gyrA* and *parC* within the quinolone-resistance-determining regions [60]. However, porin loss or altered protein expression was not experimentally confirmed through proteomic analysis, membrane fraction studies, or transcriptional assays. These findings therefore represent putative genotype–phenotype associations rather than functionally validated resistance mechanisms. Some observed substitutions may represent lineage-associated polymorphisms rather than causal resistance determinants [61].

The virulence gene repertoire of Kq1223 was conserved but limited. Core genes included *ompA*, *entB*, and *fepC*, associated with adhesion, iron acquisition, and serum resistance [51]. No hypervirulence loci such as yersiniabactin (*ybt*) or aerobactin (*iuc*) were detected, consistent with a non-hypervirulent, opportunistic phenotype. Elements of the *ecp* operon (*ykgK/ecpR* and *yagZ/ecpA*) suggest moderate biofilm formation and epithelial adherence, potentially supporting survival in hospital environments without conferring invasive capability. These findings are consistent with previous studies showing that *K. quasipneumoniae* generally lacks siderophore-mediated virulence mechanisms characteristic of invasive *K. pneumoniae* strains [51, 62, 63].

A complete Type I-E CRISPR-Cas system was also identified in Kq1223. Although CRISPR-Cas systems have been proposed to influence horizontal gene transfer and plasmid acquisition in Klebsiella spp., no functional assays or spacer–protospacer matching analyses were performed in this study. Therefore, the biological activity of the CRISPR-Cas system and its potential relationship to the apparent absence of identifiable plasmid replicons remain speculative. Furthermore, the absence of known plasmids does not exclude the possibility of prior horizontal gene transfer events or acquisition of other mobile genetic elements not detected in the present analysis and cannot be directly attributed to CRISPR activity [64].

Phylogenomic analysis confirmed the placement of Kq1223 within the *K. quasipneumoniae subsp. quasipneumoniae* clade, distinct from *K. pneumoniae sensu stricto* and *K. variicola*, with strong bootstrap support based on 100 conserved core genes. Kq1223 clustered separately from currently available publicly accessible genomes and showed closest relatedness to isolates HKU6 (Hong Kong) and UCICRE14 (USA). However, given that the present study is based on a single isolate, these findings should not be interpreted as evidence of a distinct regional lineage or broader evolutionary trend. Instead, Kq1223 may represent an under-sampled genomic variant within the currently limited global collection of *K. quasipneumoniae* genomes.

Future studies incorporating larger numbers of isolates, comparative genomics, pan-genome analyses, accessory genome profiling, and longitudinal surveillance will be necessary to better define the population structure, resistance evolution, and genomic diversity of *K. quasipneumoniae* in South Asia and globally.

This study has several limitations. First, the genomic analysis was based on a single clinical isolate, which limits broader epidemiological interpretation and prevents assessment of species prevalence, transmission dynamics, or regional population structure. Second, the identified resistance-associated mutations were inferred from genomic analyses and existing literature without functional validation through complementation assays, transcriptomic studies, proteomics, or lipid A characterization. Third, comparative genomic analyses such as accessory genome profiling, pan-genome analysis, and detailed evaluation of mobile genetic elements were beyond the scope of the present study. Finally, the absence of detectable plasmid replicons does not definitively exclude the presence of uncharacterized or low-copy mobile genetic elements. Consequently, the findings should be interpreted as preliminary genomic observations that require confirmation through expanded sampling and functional investigation.

## Conclusion

This study presents a whole-genome–based characterization of a colistin non-susceptible *K. quasipneumoniae subsp. quasipneumoniae* (isolate Kq1223) recovered from a urinary tract infection in a patient with underlying chronic conditions in Pakistan, highlighting the utility of WGS for accurate species identification within the KpSC and for the characterization of putative antimicrobial resistance mechanisms. No known plasmid replicons were detected, and the resistome was predominantly chromosomally encoded; however, the absence of detectable plasmids does not exclude the presence of uncharacterized mobile genetic elements. The genomic features identified are consistent with previously described chromosomal resistance mechanisms in Klebsiella spp., although their functional contribution was inferred and not experimentally validated. Given that this study is based on a single isolate, the findings should be interpreted as exploratory and not representative of broader epidemiological patterns or population structure. While Kq1223 clusters separately from currently available genomes, this observation may reflect limited sampling rather than evidence of a distinct regional lineage or evolutionary trajectory. Overall, these findings provide preliminary genomic insight into *K. quasipneumoniae* from an underrepresented region and underscore the need for expanded genomic surveillance and larger datasets to better define its diversity, resistance mechanisms, and clinical relevance.

## Supplementary Information

Below is the link to the electronic supplementary material.

Supplementary Material 1

## Data Availability

Raw sequence data for the *K. quasipneumoniae* subsp. *quasipneumoniae* strain (Kq1223) has been deposited in NCBI GenBank under BioProject accession number PRJNA1306097 and BioSample accession number SAMN50623229.
