# Supplementary material for: Whole-genome characterization of a colistin-resistant Klebsiella quasipneumoniae subsp. quasipneumoniae isolate from a urinary tract infection in Peshawar, Pakistan
Source: Braz J Microbiol. 2026 Jun 10;57(1):169. doi: 10.1007/s42770-026-01982-0 (PMC13253921; doi:10.1007/s42770-026-01982-0)
Supplement: Supplementary file 1 — Supplementary Material 1 [file 42770_2026_1982_MOESM1_ESM.docx]

## Whole-Genome Characterization of a Colistin-Resistant *Klebsiella quasipneumoniae subsp. quasipneumoniae* Isolate from a Urinary Tract Infection in Peshawar, Pakistan

Aiman Waheed^a^*,* Sumera Afzal^a^*,* Sajjad Ahmad^b^*,* Taj Ali Khan^b,c*^*,* Taane G. Clark^d,e^*^,^*^*^

1. Center of Biotechnology and Microbiology*,* University of Peshawar [aimu.waheed786@gmail.com](mailto:aimu.waheed786@gmail.com)*,* [drsumera@uop.edu.pk](mailto:drsumera@uop.edu.pk)
2. Institute of Pathology and Diagnostic Medicine (IPDM)*,* Khyber Medical University Peshawar*,* Pakistan*,* [tajalikhan.ibms@kmu.edu.pk](mailto:tajalikhan.ibms@kmu.edu.pk) *,* [sajjadahmad.ibms@kmu.edu.pk](mailto:sajjadahmad.ibms@kmu.edu.pk)
3. Public Health Reference Laboratory*,* Khyber Medical University*,* Peshawar*,* Pakistan.
4. Faculty of Infectious and Tropical Diseases*,* London School of Hygiene and Tropical Medicine*,* London*,* UK. [taane.clark@lshtm.ac.uk](mailto:taane.clark@lshtm.ac.uk)
5. Faculty of Epidemiology and Population Health*,* London School of Hygiene and Tropical Medicine*,* London*,* UK.

**Corresponding Authors:**

Prof. Taj Ali Khan, Prof. Taane G. Clark

**Supplementary Materials**

**Table S1.** Baseline demographic and clinical characteristics of *Klebsiella* infected patients

| Characteristics | Total Cohort (N = 64) |
| --- | --- |
| Age, years | 57 (45–68) |
| Age groups | |
| 18–39 years | 12 (18.8%) |
| 40–59 years | 26 (40.6%) |
| ≥60 years | 26 (40.6%) |
| Gender | |
| Male | 40 (62.5%) |
| Female | 24 (37.5%) |
| District of Origin | |
| Mardan | 16 (25.0%) |
| Peshawar | 12 (19.0%) |
| Swabi | 8 (12.5%) |
| Other Districts | 28 (43.5%) |
| Ethnic Background | |
| Pashtun | 46 (71.9%) |
| Hindkowan | 10 (15.6%) |
| Hazara | 5 (7.8%) |
| Other (e.g., Punjabi, Afghans) | 3 (4.7%) |
| Comorbid Conditions | |
| Diabetes Mellitus | 22 (34.4%) |
| Chronic Kidney Disease | 14 (21.9%) |
| Hypertension | 13 (20.3%) |
| Chronic Liver Disease | 8 (12.5%) |
| Immunosuppression | 7 (10.9%) |
| Care Setting at Presentation | |
| Inpatient/Ward | 47 (73.4%) |
| Outpatient Department (OPD) | 17 (26.6%) |
| Infection Type | |
| Skin and Soft Tissue Infection (SSTI) | 22 (34.4%) |
| Wound Infection | 18 (28.1%) |
| Urinary Tract Infection (UTI) | 9 (14.1%) |
| Abscess | 7 (10.9%) |
| Cellulitis | 5 (7.8%) |
| Septicemia | 3 (4.7%) |

**Table S2.** Genome assembly, annotation and proteomic features of *K. quasipneumoniae* (Kq1223)

| Features | Value |
| --- | --- |
| Assembly Metrics | |
| Total Contigs | 98 |
| Genome Length | 5,368,757 bp |
| GC Content | 57.96% |
| Contig N50 | 365,211 bp |
| Contig L50 | 5 |
| Chromosomes | 0 |
| Plasmids | 0 |
| Annotation Metrics | |
| Coding Sequences (CDS) | 5,227 |
| tRNA Genes | 86 |
| rRNA Genes | 13 |
| Repeat Regions | 9 |
| Partial CDS | 0 |
| Miscellaneous RNA | 0 |
| Proteomic Features | |
| Hypothetical Proteins | 720 |
| Proteins with Functional Assignments | 4,507 |
| Proteins with EC Number Assignments | 1,414 |
| Proteins with GO Term Assignments | 1,170 |
| Proteins Associated with Metabolic Pathways | 1,037 |
| Proteins in PATRIC Genus-Specific Families (PLfam) | 5,034 |
| Proteins in PATRIC Cross-Genus Families (PGfam) | 5,054 |

**Table S3**. Publicly available *Klebsiella quasipneumoniae subsp. quasipneumoniae* genomes included in the phylogenomic analysis

| Strain | GenBank Accession | Isolation Source | BioProject Accession | Collection Year | Country |
| --- | --- | --- | --- | --- | --- |
| UCICRE14 | AYIC00000000 | Sputum | PRJNA202000 | 2014 | USA |
| 190624049722 | JAGKIS000000000 | Catheter | PRJNA717735 | 2019 | Brazil |
| 190624049721 | JAGKIT000000000 | Bloodstream | PRJNA717735 | 2019 | Brazil |
| 191106182516 | JAGKIZ000000000 | Urine | PRJNA717735 | 2019 | Brazil |
| 191106182515 | JAGKJA000000000 | Bloodstream | PRJNA717735 | 2019 | Brazil |
| 191106182511 | JAGKJG000000000 | Bloodstream | PRJNA717735 | 2017 | Brazil |
| 191106182512 | JAGKJF000000000 | Bloodstream | PRJNA717735 | 2017 | Brazil |
| SBH035 | JALIFN000000000 | Urine | PRJNA822851 | 2021 | China |
| HKp70 | JAXSAD000000000 | Blood | PRJNA1052047 | 2020 | Canada |
| HKU12 | JAVBDO000000000 | Blood | PRJNA1003408 | 2016 | Hong Kong |
| HKU6 | JAVBFI000000000 | Blood | PRJNA1003408 | 2016 | Hong Kong |
| 191106182520 | JAIQUN000000000 | Hospital environment | PRJNA717735 | 2019 | Brazil |
| A73113 | JBDNCE000000000 | Blood | PRJNA1112783 | 2018 | Brazil |
| 1972/2978 | PTHR00000000 | Miscellaneous body fluid | PRJNA433394 | 2015 | USA |
| HU292 | JBCGPR000000000 | Sputum | PRJNA1101749 | 2024 | China |
| HU272 | JBCGPO000000000 | Secretion | PRJNA1101749 | 2024 | China |
| HU264 | JBCGPN000000000 | Urine | PRJNA1101749 | 2024 | China |
| HU52 | JBCGOU000000000 | Secretion | PRJNA1101749 | 2024 | China |
| HU51 | JBCGOT000000000 | Secretion | PRJNA1101749 | 2024 | China |
| HU111 | JBCGNY000000000 | Sputum | PRJNA1101749 | 2024 | China |
| GX19 | JBCGMY000000000 | Cerebrospinal fluid | PRJNA1101749 | 2024 | China |
| GX31 | JBCGNC000000000 | Urine | PRJNA1101749 | 2024 | China |
| CRK0052 | NFAE00000000 | Blood | PRJNA339843 | 2014 | USA |
| FI_HV_2014 | LGAL00000000 | Blood | PRJNA288524 | 2014 | Italy |
| PO1285 | NFWG00000000 | Blood | PRJNA351846 | 2010 | Nigeria |
| AK_SD_007 | NFYR00000000 | Blood | PRJNA351846 | 2014 | Nigeria |
| KPN169 | JAJRKZ000000000 | Blood | PRJNA788509 | NA | USA |
| KPN1858 | JAJHNT000000000 | Urine | PRJNA777643 | 2020 | Australia |
| IMP41 | CAIZUA000000000 | Not available | PRJEB38818 | 2019 | United Kingdom |
| JXR167 | JALKGY000000000 | Not available | PRJNA674484 | 2015 | China |
| JXR166 | JALKGZ000000000 | Not available | PRJNA674484 | 2015 | China |
| KKP036 | JAJHYI000000000 | Skin and soft tissue | PRJNA777842 | 2020 | Kenya |
| MRSN825396 | JAJHYT000000000 | Urinary tract | PRJNA777842 | 2016 | Kenya |
| SCKQ020041 | NWEK00000000 | Not available | PRJNA353728 | 2016 | China |
| XJ-190 | JAOUTP000000000 | Not available | PRJNA887693 | 2015 | China |
| HN-10 | JAOYSG000000000 | Not available | PRJNA887693 | 2015 | China |
| 2028 | JAOUUQ000000000 | Not available | PRJNA887693 | 2020 | China |
| R110 | JAOUVO000000000 | Not available | PRJNA887693 | 2018 | China |
| KP8 | JAVIFZ000000000 | Sputum | PRJNA1009584 | 2018 | China |

**Figure S1:** Krona Pie Chart showing the taxonomy of Kq1223
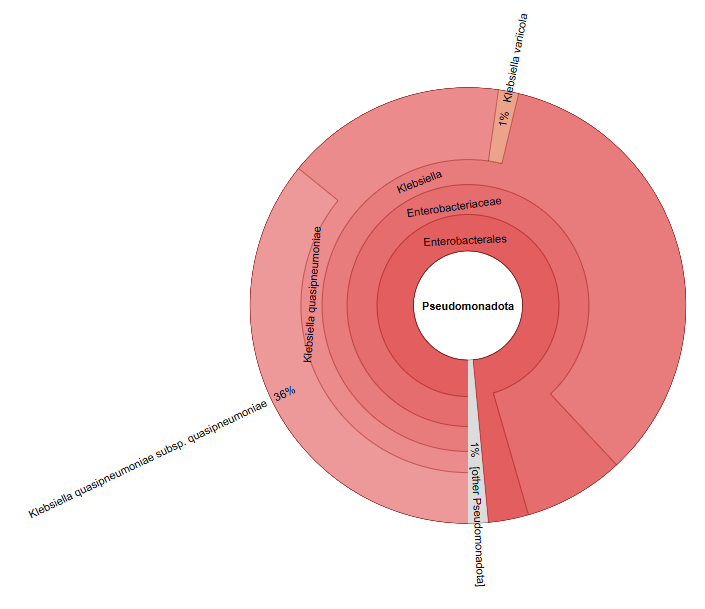


**Figure S2.** Core genome phylogenetic tree of Kq1223 and publicly available *K. quasipneumoniae subsp quasipneumoniae* genomes in BV-BRC**
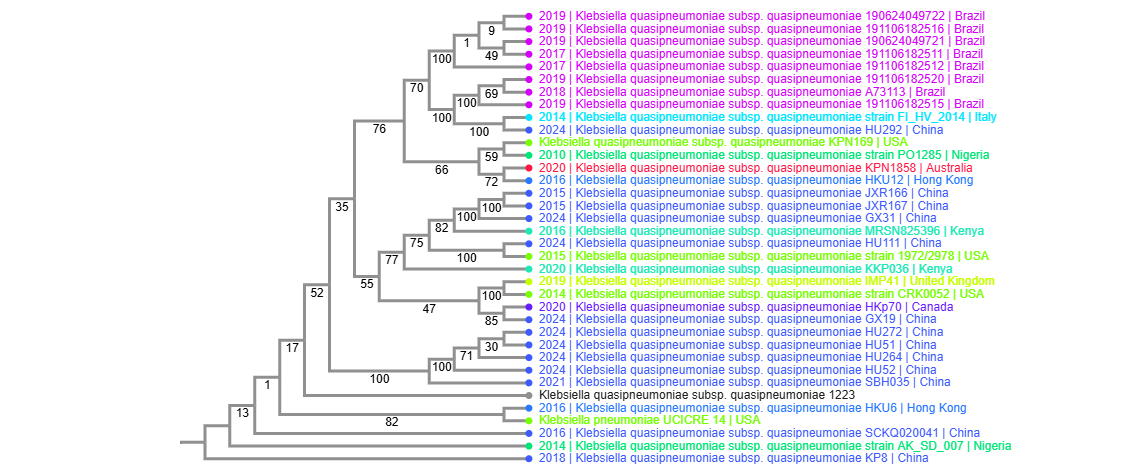
**
